# Genome-Wide Identification and Expression Profiling of ATP-Binding Cassette (ABC) Transporter Gene Family in Pineapple (*Ananas comosus* (L.) Merr.) Reveal the Role of *AcABCG38* in Pollen Development

**DOI:** 10.3389/fpls.2017.02150

**Published:** 2017-12-19

**Authors:** Piaojuan Chen, Yi Li, Lihua Zhao, Zhimin Hou, Maokai Yan, Bingyan Hu, Yanhui Liu, Syed Muhammad Azam, Ziyan Zhang, Zia ur Rahman, Liping Liu, Yuan Qin

**Affiliations:** State Key Laboratory of Ecological Pest Control for Fujian and Taiwan Crops, Fujian Provincial Key Laboratory of Haixia Applied Plant Systems Biology, Center for Genomics and Biotechnology, College of Life Science, Fujian Agriculture and Forestry University, Fuzhou, China

**Keywords:** pineapple, sexual reproduction, *ABC* genes, expression profile, pollen abortion

## Abstract

Pineapple (*Ananas comosus L*.) cultivation commonly relies on asexual reproduction which is easily impeded by many factors in agriculture production. Sexual reproduction might be a novel approach to improve the pineapple planting. However, genes controlling pineapple sexual reproduction are still remain elusive. In different organisms a conserved superfamily proteins known as ATP binding cassette (ABC) participate in various biological processes. Whereas, till today the *ABC* gene family has not been identified in pineapple. Here 100 *ABC* genes were identified in the pineapple genome and grouped into eight subfamilies (5 *ABCAs*, 20 *ABCB*s, 16 *ABCCs*, 2 *ABCDs*, one *ABCEs*, 5 *ABCFs*, 42 *ABCGs* and 9 *ABCIs*). Gene expression profiling revealed the dynamic expression pattern of *ABC* gene family in various tissues and different developmental stages. *AcABCA5, AcABCB6, AcABCC4*, *AcABCC7*, *AcABCC9*, *AcABCG26*, *AcABCG38* and *AcABCG42* exhibited preferential expression in ovule and stamen. Over-expression of *AcABCG38* in the *Arabidopsis* double mutant *abcg1-2abcg16-2* partially restored its pollen abortion defects, indicating that *AcABCG38* plays important roles in pollen development. Our study on *ABC* gene family in pineapple provides useful information for developing sexual pineapple plantation which could be utilized to improve pineapple agricultural production.

## Introduction

The ATP-binding cassette (ABC) gene family is one of the largest expressed gene subfamilies (Campa et al., 2008). Most ABC transporter subfamilies had highly conservative amino acid sequence domains: the nucleotide-binding domain (NBDs) and the transmembrane domains (TMDs) including five or six helices (Higgins and Linton, 2004). The NBD provides energy by hydrolyzing ATP, and the TMDs determines the substrate specificity (Schneider and Hunke, 1998). NBDs contains three exceptional motifs: the signature motif, the Walker A motif and the Walker B motif. The signature motif is unique to ABC proteins while the Walker A and Walker B motif are responsible for nucleotide binding (Davidson et al., 2008). ABC transporter proteins have been classified into two categories based on the length of structural domain: full-sized transporters (two NBDs and two TMDs) and half-sized transporters (one NBD) (Davidson et al., 2008). Protein structures were closely associated with function. The *ABCE* and *ABCF* subfamilies lack of TMDs domain and do not function as transporter. For example, the first human and mammalian ABC protein ABC50 which does not contain TMDs, were endowed with ribosome assembly (Richard et al., 1998; Tyzack et al., 2000). *ABCE* (*Y39E4B.1*) had been reported for regulating transcription and translation in eukaryotes (Zhao et al., 2004).

The majority of *ABC* family members are involved in transporting a variety of compounds through biological membranes, such as lipids, suberin, steroids, irons, amino acids and other metabolic substances (Klein et al., 1999). Disruption of ABC transporter proteins has huge impact on bacterial physiology such as toxic effect (Henderson and Payne, 1994). The ABC transporters also participated in plant development. In *Arabidopsis*, auxin regulate almost every biological processes from early embryonic development to leaf senescence (Paponov et al., 2005). AtPGP1 and AtPGP19, two of *Arabidopsis* multidrug-resistance-like ABC transporters control polar auxin transport and the double mutant *atpgp1-1atpgp19-1* exhibits epinastic growth and small inflorescence size (Geisler et al., 2003). It was recently shown that *AtABCG1* and *AtABCG16* implicated in the integrity of exine and nexine of pollen wall and the formation of pollen-derived intine layer (Yadav and Reed, 2014). The microspores of double mutants *abcg1abcg16* exhibit defects in pollen mitosis in postmeiotic stages of male gametophyte development as well as lack of intact nexine and intine pollen layers in *Arabidopsis* (Yim et al., 2016).

Pineapple (*Ananas comosus L*.), a perennial monocot from the Bromeliaceae family, is one of the famous tropical and edible flavorful fruits. Pineapple is usually diploid (2n = 50) and the haploid genome size is estimated to be 526 Mb (Arumuganathan and Earle, 1991). In agriculture production, pineapple planting is impeded by abiotic and/or biotic factors, such as cold, fusarium wilt, and other diseases (Pujol and Kado, 2000; Peckham et al., 2010; Wang et al., 2014; Santos et al., 2016). In addition, pineapple is one of the self-incompatible species, and cultivated mostly by vegetative propagation. The pineapple varieties resulted by vegetative propagation greatly reduces fruit quality and heterogeneity (Fassinou Hotegni et al., 2015). These disadvantages of asexual cultivation could be effectively improved by sexual propagation. However, sexual propagation through clearing harmful mutations could improve the fruit quality of generations, which was more adaptive to effective evolutionary responses to natural selection (Otto, 2009; Neiman and Schwander, 2011; Levitis et al., 2017). Recent study shows that pollen development is crucial for sexual reproduction in plants and the quantity and quality of pollen received determines pollen limitation (Aizen and Harder, 2007; Rodger and Ellis, 2016). Overall, there is a serous limitation in pineapple vegetative propagation and sexual reproduction could become novel approach to overcome these problems. In view of the widely function were involved in reproduction development for ABC transporter proteins but how AcABC proteins function in pineapple reproduction is unknown, genome-wide identification, characterization and function study of the ABC transporter gene family in pineapple would become very meaningful.

Recently, sequencing and genome assembly of the entire genome of pineapple has been completed (Ming et al., 2015), which made it possible to systematically study of the *ABC* subfamily in pineapple. In this study, we performed the analyses of gene phylogeny, gene structure, and expression profiles of ABC proteins in different reproductive organs and different developmental stages in pineapple. We provided comprehensive information of ABC proteins in pineapple and determined the critical role of the stamen enriched gene *AcABCG38* in pollen development. Present work may contribute to improve pineapple production though sexual reproduction.

## Materials and Methods

### Identification of *ABC* Transporter Genes in Pineapple Genome

The *AcABC* protein and genome sequence were downloaded from Phytozome v12.1^1^. To identify the *AcABC* genes, the HMMER3.0^2^ was used with default parameters settings to search the proteins sequences containing PFAM ABC domain (PF00005) (Verrier et al., 2008). To achieve accuracy in our analysis, we further used NCBI-CDD with *E*-value threshold 0.01^3^ to analyze the conservative sequences and to remove any sequences which lack ABC annotation (Zhang et al., 2017). Isoelectric point (PI) and molecular weight (MW) of the AcABC family member proteins were obtained using ExPAsy website^4^ (**Table 1**).

**Table 1:** The *ABC* gene family in pineapple.

Group	Transcipt ID	Gene name	Topology	Ch	Length(aa)	MW(Kd)	pI	Main loca.
Subfamily A, 5 members								
AOH	Aco007263.1	AcABCA1	(TMD-NBD)_2_	LG23	1885	209.74	7.28	fruit.S7
ATH	Aco028137.1	AcABCA2	TMD-NBD	scaffold_1582	967	107.06	7.81	Flower
	Aco006844.1	AcABCA3	TMD-NBD	LG01	961	106.43	6.95	Stamen.S5
	Aco006845.1	AcABCA4	TMD-NBD	LG01	890	99.17	8.62	Ovule.S7
	Aco014363.1	AcABCA5	TMD-NBD	LG05	947	106.5	8.76	Flower
Subfamily B, 18 members								
MDR	Aco010196.1	AcABCB1	(TMD-NBD)_2_	LG25	1252	144.98	9.32	Ovule.S1
	Aco012952.1	AcABCB2	(TMD-NBD)_2_	LG03	1216	139.29	9.18	Petal.S3
	Aco027643.1	AcABCB3	TMD-NBD-TMD	scaffold_777	992	107.44	8.91	Root
	Aco016500.1	AcABCB4	(TMD-NBD)_2_	LG11	1613	108.46	9.03	Stamen.S4
	Aco022110.1	AcABCB5	(TMD-NBD)_2_	LG04	1294	140.81	7.89	fruit.S4
	Aco001135.1	AcABCB6	(TMD-NBD)_2_	LG02	1239	136.8	8.75	fruit.S4
	Aco018951.1	AcABCB7	(TMD-NBD)_2_	LG12	1258	140.34	8.97	Stamen.S2
	Aco006827.1	AcABCB8	(TMD-NBD)_2_	LG01	1295	135.1	9.03	Stamen.S3
	Aco019592.1	AcABCB9	(TMD-NBD)_2_	LG10	978	138.8	8.68	Flower
	Aco013278.1	AcABCB10	(TMD-NBD)_2_	LG24	1395	139.04	8.69	Ovule.S6
	Aco016496.1	AcABCB11	(TMD-NBD)2-NBD	LG11	1407	174.98	6.21	Root
	Aco022325.1	AcABCB12	(TMD-NBD)_2_	LG19	1263	135.39	8.81	fruit.S1
	Aco003987.1	AcABCB19	(TMD-NBD)_2_	LG15	645	151.04	6.27	Stamen.S3
	Aco001486.1	AcABCB20	(TMD-NBD)_2_	LG18	1270	155.88	6.4	Stamen.S1
	Aco008863.1	AcABCB13	(TMD-NBD)_2_	LG09	1340	132.98	8.9	Stamen.S2
TAP	Aco007808.1	AcABCB14	TMD-NBD	LG21	1653	70.41	7.98	Stamen.S3
	Aco017569.1	AcABCB15	TMD-(NBD)2-TMD-NBD	LG09	756	179.38	6.29	fruit.S4
	Aco015743.1	AcABCB16	TMD_2_-NBD	LG09	741	82.03	9.05	Ovule.S4
	Aco017326.1	AcABCB17	TMD-NBD	LG01	755	82.29	8.86	Ovule.S2
ATM	Aco011166.1	AcABCB18	TMD-NBD	LG04	309	82.67	9.32	fruit.S7
Subfamily C, 16 members								
	Aco009750.1	AcABCC1	(TMD-NBD)_2_	LG10	1545	170.6	7.71	Flower
	Aco014828.1	AcABCC2	(TMD-NBD)_2_	LG14	1543	173.73	6.83	Ovule.S1
	Aco027927.1	AcABCC3	(TMD-NBD)_2_	scaffold_1613	1544	170.76	8.16	Stamen.S5
	Aco019200.1	AcABCC4	(TMD-NBD)_2_	LG13	1404	154.1	6.18	Stamen.S4
	Aco010163.1	AcABCC5	(TMD-NBD)_2_	LG25	1522	168.42	8.27	Flower
	Aco019507.1	AcABCC6	(TMD-NBD)_2_	LG07	2068	228.97	7.1	Ovule.S3
	Aco009944.1	AcABCC7	(TMD-NBD)_2_	LG10	1463	163.87	8.43	Stamen.S4
	Aco015193.1	AcABCC8	(TMD-NBD)_2_	LG05	1463	163.62	8.82	fruit.S3
	Aco000353.1	AcABCC9	(TMD-NBD)_2_	LG12	1491	166.33	7.94	Stamen.S5
	Aco005539.1	AcABCC10	(TMD-NBD)_2_	LG11	1485	164.82	8.42	Stamen.S1
	Aco006625.1	AcABCC11	(TMD-NBD)_2_	LG01	1583	177.22	8.71	Ovule.S2
	Aco025745.1	AcABCC12	NBD	LG01	348	39.08	5.29	Ovule.S1
	Aco016941.1	AcABCC13	(TMD-NBD)_2_	LG02	1285	143.55	6.29	Petal.S3
	Aco017858.1	AcABCC14	(TMD-NBD)_2_	LG23	1261	140.25	6.04	Sepal.S2
	Aco012910.1	AcABCC15	(TMD-NBD)_2_	LG03	1514	169.94	8.6	Leaf
	Aco016196.1	AcABCC16	TMD-NBD	LG17	309	33.65	9.69	Ovule.S4
Subfamily D, 2 members								
	Aco023353.1	AcABCD1	(TMD-NBD)_2_	LG18	1342	149.88	9.16	Stamen.S5
	Aco010685.1	AcABCD2	TND-NBD	LG10	750	83.8	8.48	Stamen.S3
Subfamily E, 1 members								
	Aco010811.1	AcABCE1	NBD_2_	LG10	605	68.22	8.04	Stamen.S5
Subfamily F, 5 members								
	Aco010200.1	AcABCF1	NBD_3_	LG25	601	67.25	6.07	Petal.S3
	Aco006898.1	AcABCF2	NBD_3_	LG22	603	67.03	6.37	Leaf
	Aco009295.1	AcABCF3	NBD_3_	LG22	719	79.99	5.92	Stamen.S1
	Aco018812.1	AcABCF4	NBD_2_	LG13	742	81.44	5.87	Stamen.S4
	Aco000176.1	AcABCF5	NBD_3_	LG12	717	79.54	6.07	fruit.S2
Subfamily G,42 members								
PDR	Aco005449.1	AcABCG1	TMD-NBD-TMD_2_	LG11	1234	140.02	7.35	Sepal.S2
	Aco005451.1	AcABCG2	(NBD-TMD)_2_	LG11	1393	157.13	8.66	Stamen.S1
	Aco027149.1	AcABCG6	(NBD-TMD)_2_	scaffold_1231	1419	159.96	8.93	Sepal.S3
	Aco009786.1	AcABCG8	(NBD-TMD)_2_	LG10	1347	151.89	8.83	Flower
	Aco006783.1	AcABCG12	(NBD-TMD)_2_	LG01	1456	164.5	8.47	fruit.S5
	Aco006142.1	AcABCG13	(NBD-TMD)_2_	LG16	1476	166.35	6.59	Sepal.S1
	Aco023493.1	AcABCG19	(NBD-TMD)_2_	LG01	1416	160.42	7.85	Stamen.S4
	Aco015369.1	AcABCG20	(NBD-TMD)_2_	LG23	1449	161.94	8.62	Stamen.S5
	Aco021666.1	AcABCG21	(NBD-TMD)_2_	LG07	1282	145.07	8.65	fruit.S1
	Aco009791.1	AcABCG31	(NBD-TMD)_2_	LG10	1452	165.01	8.77	Ovule.S7
	Aco006143.1	AcABCG36	(NBD-TMD)_2_	LG16	1410	158.85	6.51	Ovule.S4
	Aco014323.1	AcABCG39	(NBD-TMD)_2_	LG05	1450	164.01	7.54	Sepal.S2
	Aco013658.1	AcABCG41	(NBD-TMD)_2_	LG13	1452	164.64	7.72	Stamen.S3
	Aco015172.1	AcABCG42	NBD2-TMD-NBD-TMD	LG05	1584	177.88	8.91	Stamen.S4
WBC	Aco010405.1	AcABCG3	NBD-TMD	LG03	727	80.78	9.09	Root
	Aco030876.1	AcABCG4	NBD-TMD	scaffold_1304	612	65.76	8.91	Sepal.S3
	Aco011308.1	AcABCG5	NBD-TMD	LG01	610	66.69	9.39	fruit.S5
	Aco021246.1	AcABCG7	NBD-TMD	LG10	691	75.26	8.96	fruit.S1
	Aco009154.1	AcABCG9	NBD-TMD	LG09	642	70.19	8.61	Stamen.S3
	Aco006105.1	AcABCG10	NBD-TMD	LG16	591	64.95	9.14	Root
	Aco010632.1	AcABCG11	NBD-TMD	LG07	723	80.1	9.18	Stamen.S2
	Aco023881.1	AcABCG14	NBD	LG20	88	9.32	10.37	/
	Aco007271.1	AcABCG15	NBD-TMD	LG23	677	75.25	9.59	Ovule.S1
	Aco020513.1	AcABCG16	NBD	LG01	283	31.43	11.09	Leaf
	Aco010077.1	AcABCG17	NBD-TMD	LG25	714	78.57	8.54	Ovule.S4
	Aco001046.1	AcABCG18	Arf-NBD-TMD	LG02	896	100.18	8.27	fruit.S7
	Aco021024.1	AcABCG22	NBD-TMD	LG15	743	81.87	9.15	Sepal.S1
	Aco002328.1	AcABCG23	NBD-TMD	LG04	612	68.93	6.81	Root
	Aco023879.1	AcABCG24	NBD	LG20	201	21.67	9.49	Root
	Aco003255.1	AcABCG25	NBD-TMD	LG17	605	65.4	9.38	Ovule.S7
	Aco023619.1	AcABCG26	NBD-TMD	LG19	641	71.96	9.36	Stamen.S4
	Aco013155.1	AcABCG27	NBD-TMD	LG24	601	66.02	9.42	Stamen.S3
	Aco006887.1	AcABCG28	NBD	LG22	1089	120.53	9.12	Ovule.S5
	Aco007658.1	AcABCG29	NBD-TMD	LG08	721	79.81	8.84	Flower
	Aco001237.1	AcABCG30	NBD-TMD	LG02	728	80.13	7.59	Flower
	Aco031846.1	AcABCG32	NBD-TMD	scaffold_3635	372	39.56	8.97	Stamen.S1
	Aco004405.1	AcABCG33	NBD-TMD	LG05	605	64.96	8.82	Sepal.S4
	Aco000542.1	AcABCG34	NBD-TMD	LG12	642	70.76	10.47	Leaf
	Aco010996.1	AcABCG35	NBD-TMD	LG04	707	77.84	8.43	Sepal.S2
	Aco005255.1	AcABCG37	NBD-TMD	LG07	720	79.28	8.97	Petal.S3
	Aco001503.1	AcABCG38	NBD-TMD	LG18	794	87.24	9.1	Stamen.S3
	Aco019952.1	AcABCG40	NBD	LG08	861	96.25	9.2	Stamen.S4
Subfamily I, 9 members								
	Aco008459.1	AcABCI1	NBD	LG19	224	24.87	9.69	Petal.S3
	Aco028846.1	AcABCI2	NBD	scaffold_627	301	32.04	8.48	Stamen.S1
	Aco018474.1	AcABCI3	NBD	LG21	431	48.82	7.17	Stamen.S1
	Aco005118.1	AcABCI4	NBD	LG07	283	30.25	8.56	Sepal.S1
	Aco001741.1	AcABCI5	NBD	LG18	307	33.24	8.68	Stamen.S2
	Aco007380.1	AcABCI6	NBD	LG23	326	34.26	6.62	Stamen.S1
	Aco006975.1	AcABCI7	NBD	LG22	291	32.74	5.48	fruit.S4
	Aco030323.1	AcABCI8	NBD	scaffold_1361	312	32.85	6.4	Petal.S2
	Aco004516.1	AcABCI9	NBD	LG05	350	38.23	8.2	fruit.S3


*Columns one to nine show the subgroup names, gene transcript ID, gene name, proteins gross topology (domain, orientation, number of modules, from “-NH3” to “-COOH”), chromosome location, numbers of amino acid residues in the gene translation product, proteins size, protein isoeletric point and main expression location.*

### Phylogenetic Analysis

To understand the phylogenetic relationship of ABC proteins between pineapple and *Arabidopsis*, all the identified ABC amino acid sequences of pineapple and *Arabidopsis* were used to construct the phylogenetic tree. AcABC and AtABC protein sequences were aligned using MAFFT with default parameters^5^. Then, FastTree software was used to establish phylogenetic tree using 1,000 resamples, and constructed by maximum-likelihood using the JTT + CAT model. FastTree provides local support values by the Shimodaira-Hasegawa (SH) test, the resulting support values were closely correlated with the traditional bootstrap (*r* = 0.975) (Price et al., 2010; Chen et al., 2017). The local support values below 60 are hidden in the constructed tree. All amino acid sequences were used for phylogenetic and alignments analysis in the study were supplied in **Supplementary Datasets S1**, **S2**.

### Gene Structure Analysis and Conserved Motif Identification

The exon–intron characteristics of the *ABC* genes family were exhibited using the Gene Structure Display Server^6^ (Guo et al., 2007). Through a comparison with the full-length predicted coding sequence (CDS). The motifs of the AcABCG proteins were determined with the appropriate number of motifs using the MEME program^7^. The lower *E*-value (the most statistically significant), the more accurate of expected motifs.

### Plant Materials and Growth Conditions

The wild-type *Arabidopsis* background Col-0 (CS60000) and the T-DNA insertions of *AtABCG1* (*abcg1-1*: SALK_061511; *abcg1-2*: SALK_055389) and *AtABCG16* (abcg16-1: SALK_087501; abcg16-2: SALK_119868C) were obtained from *Arabidopsis* Biological Resource Center (ABRC)^8^. Pineapple (*Ananas comosus)* variety MD2 was collected by Qin Lab^9^. Pineapple were grown in plastic pots with soil mix [peat moss: perlite = 2:1 (v/v)] and placed in greenhouse at about 30°C with light availability of 60–70 mMolL^-1^ photons m^-2^s^-1^ under 70% humidity with 16 h light/8 h dark photoperiod. *Arabidopsis* was grown with the conditions as described by Cai et al. (2017).

### RNA-Seq and qRT-PCR

Tissue samples of MD2 were collected from different developmental stages of ovule, petal, sepal and stamen. The criterion of different stage samples was referenced to Su et al. (2017), including seven stages of ovule (Ov1-Ov7), three stages of petal (Pe1–Pe3), five stages of stamen (St1–St5) and four stages of sepal (Se1–Se4). Collected samples were quickly stored in liquid nitrogen until total RNA extraction. The RNA was extracted following manufacturer’s protocol using RNA extraction Kit (Omega Bio-Tek, Shanghai, China). Total RNA was diluted with nuclease-free water and then mRNA was isolated, following by fragmentation, and the first and second strand cDNA synthesized. Double-stranded cDNA was then purified using 1.8× Agencourt AMPure XP Beads. Performing End Repair/dA-tail of cDNA Library followed by adaptor ligation using Blunt/TA Ligase Master Mix and diluted NEB Next Adaptor. Purifying the ligation reaction and approximate insert size was kept 25–400 bp with final library was set to 300–500 bp. Performing PCR Library construction followed by purity of the PCR reaction using Agencourt AMPure XP Beads and assessed library quality on a Bioanalyzer^®^ (Agilent high sensitivity chip) and send to company for sequencing (NEB next Ultra RNA Library Prep Kit for Illumina Biolabs). The RNA-seq data of root, leaf, flower and developing fruit were download from NCBI database^10^, and all of RNA-seq data were analyzed following Trapnell et al. (2012). Raw reads were filtered by TRIMMOMATIC v0.3 to remove the adapter sequence, the clean reads were then aligned using the Tophat software with default parameters. Alignment results were processed using Cufflinks, and FPKM values were calculated by using Cuffdiff (FC ≥ 2, FDR ≤ 0.05) for following Dai et al. (2017) analysis. *R* software was employed to construct the heat-map using FPKM values of RNA-seq for each gene. RNA-seq data were further validated by qRT-PCR analysis in different tissues (i.e., ovule Ov3, stamen St2, sepal Se3 and petal Pe3). qRT-PCR was carried out using the SYBR Premix Ex Taq II (TaKaRa, China) on a Bio-Rad Real-time PCR system (Foster, United States), and the program was: 95°C for 30 s; 40 cycles of 95°C for 5 s and 60°C for 34 s; 95°C for 15 s. Three technical replicates and at least three independent biological replicates were performed in each condition.

### Vector Construction

The full-length of coding sequence of *AcABCG38* were amplified using primers listed in **Supplementary Table S4**. The PCR fragment was cloned into the pENTR/D-TOPO vector (Invitrogen) and sequenced. The positive clone was recombined into the destination vector pGWB505 by LR reaction. The *Agrobacterium tumefaciens* (GV3101) with *AcABCG38* was used to transform into the *abcg1-2abcg16-2* double mutant and WT using a floral dip procedure (Clough and Bent, 1998). The multiple independent T1 plants were analyzed.

### Alexander Red Staining

*Arabidopsis* were fixed in FAA for 24 h, and then picked the anthers on slide, heated until the pollens were dyed in red color. The stained anthers were taken picture by microscope (Carl-Zeiss, Germany).

## Results

### Identification and Characterization of the Pineapple ABC Transporters

To identify the *ABC* gene family in pineapple, the HMMER3.0 (Eddy, 2011) was used to get ABC protein sequences containing PFAM ABC domain (PF00005) in pineapple genome database downloaded from the Phytozome v12.1. To obtain accurate *AcABC* members, the conserved sequences of AcABC were analyzed using NCBI and sequences that lacked ABC annotation were removed. Finally, a total of 100 ABC genes were identified in the pineapple genome by BLAST and phylogenetic analysis with *Arabidopsis* ABC proteins. Those genes chromosomes distribution was showed in **Supplementary Figure S1**. The 100 pineapple proteins were grouped into eight subfamilies, including 5 *ABCAs*, 20 *ABCBs* 16 *ABCCs*, 2 *ABCDs*, 1 *ABCEs*, 5 *ABCFs*, 42 *ABCGs* and 9 *ABCIs* (**Table 1**). Although the *ABCH* subfamily were ubiquitously existed in insects, fishes, echinodermata and myxomycetes (Shao et al., 2013), and it was not identified in the pineapple genomes, this result is similar to the finding of other plant species (Pang et al., 2013). The pineapple *ABC* genes were named and further classified according to the sequence similarity of *Arabidopsis ABC* genes. *AcABCAs* were subdivided into two types: *ATH* (*ABC1* homologous) and *AOH* (*ABC1* homologous), and *AcABCBs* included three subgroups: *MDR* (multidrug resistance protein), *TAP* (transporter associated with antigen processing) and *ATM* (ABC transporter of the mitochondria); *AcABCGs* were also classified into two categories: *WBC* (white-brown complex homologue) and *PDR* (pleiotropic drug resistance) (Sánchezfernández et al., 2001). The amino acids numbers ranged from 88 aa (*AcABCG14*) to 2068 aa (*AcABCC6*) with the corresponding molecular weight varied from 9.32 to 228.97 Kd. The series of information about *AcABCs* including subgroup names, gene transcript ID, numbers of amino acid residues, proteins size, protein isoelectric point and main expression location were showed in **Table 1**.

### Phylogenetic Analysis of *ABC* Family in Pineapple

**FIGURE 1:**
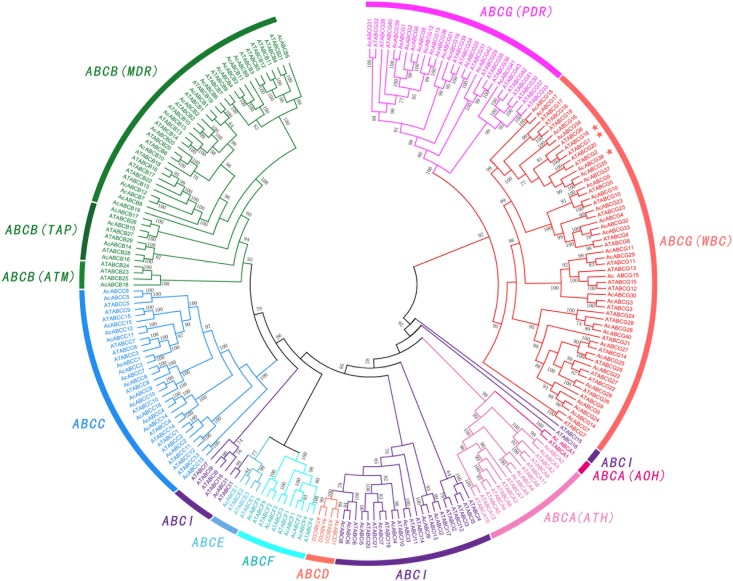
Phylogenetic relationship of *ABC* gene among Pineapple (Ac) and *Arabidopsis* (At). All the AcABC protein sequences were aligned by using *MAFFT* and phylogenetic tree was constructed using *FastTree.* Different colors represented individual subfamily. Every subfamily had another classified names: ATH (ABC1 homologous), AOH (ABC1 homologous), MDR (multidrug resistance protein), TAP (transporter associated with antigen processing), ATM (ABC transporter of the mitochondria), WBC (white-brown complex homolog), PDR (pleiotropic drug resistance). *AcABCG38*, *AtABCG1* and *AtABCG16* were indicated by asterisk.

To further understand the relationship between the *AcABCs* and *AtABCs*, full-length protein sequences of pineapple and *Arabidopsis* ABCs were aligned using MAFFT, and a combined phylogenetic tree was constructed using FastTree. The result showed that the *ABC* genes of these two species can be divided into eight subfamilies (**Figure 1**). Among those subfamilies, *ABCG* had the largest members with 42 pineapple genes and 43 *Arabidopsis* genes. *ABCB* was the second largest subgroup, containing eighteen pineapple genes and twenty-eight *Arabidopsis* genes. The smallest subgroup was *ABCD* and *ABCE*, both contain only four members: two *AcABCD* genes and two *AtABCD* genes, and one *AcABCEs* and three *AtABCEs*. According to functional characteristic, the ABCA proteins were further classified into in two distinct types with two members in ATH and 22 in AOH. Similarly, the *ABCBs* subfamily were also divided into three groups (34 *MDRs*, 8 *TAPs* and 4 *ATMs*) and *ABCGs* were divided into two types (56 *WBCs* and 29 *PDRs*). While *ABCI* subfamily was divided into three clusters in phylogenetic tree (**Figure 1**). One of which contained only two *Arabidopsis* members (*AtABCI15* and *AtABCI16*) without any pineapple members, indicating that the *ABCI* family could had undergone evolutionary divergence between dicotyledonous and monocotyledonous plants.

### Gene Structure Analysis and Conserved Motif Identification

**FIGURE 2:**
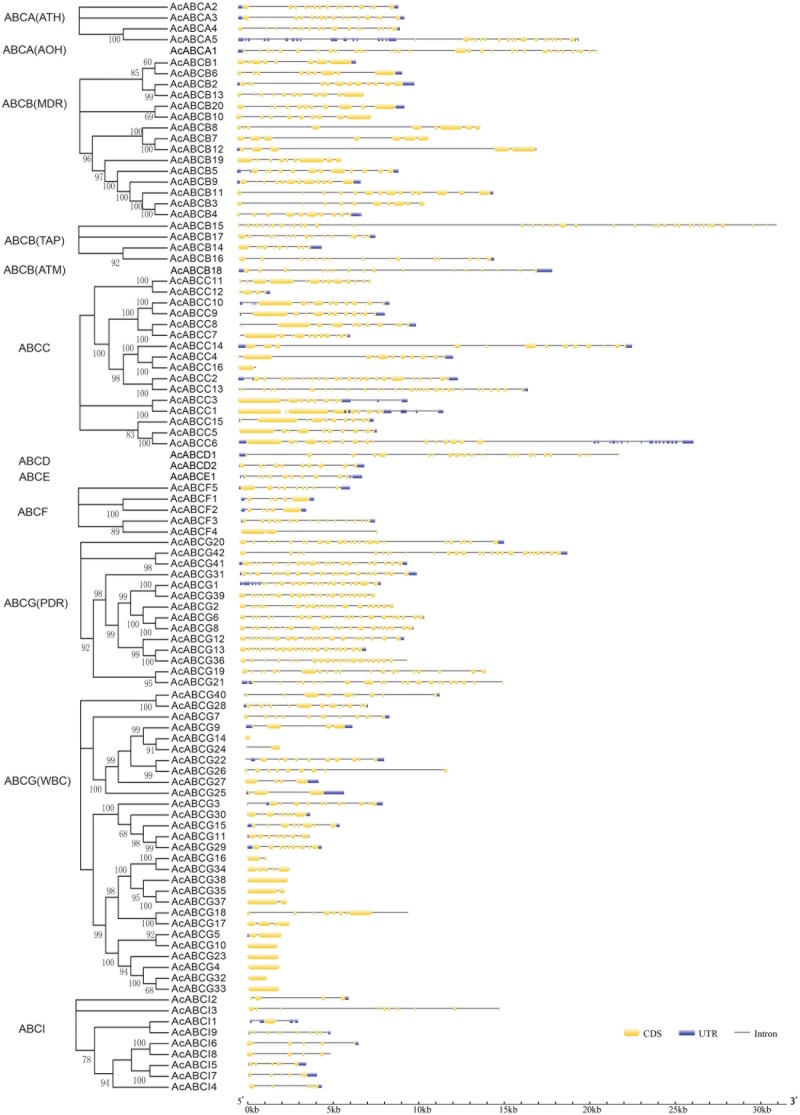
Intron–exon structure of *AcABC* genes in pineapple genome. Yellow bars indicates exon (CDS), Blue bars indicated UTR while plain lines showing introns. black lines represented introns. On the left panel is the phylogenetic trees of *ABC* transporter proteins in pineapple which was constructed by Maximum likelihood method. The numbers on the right indicate the genomic length of the corresponding genes. bp, base pair.

**FIGURE 3:**
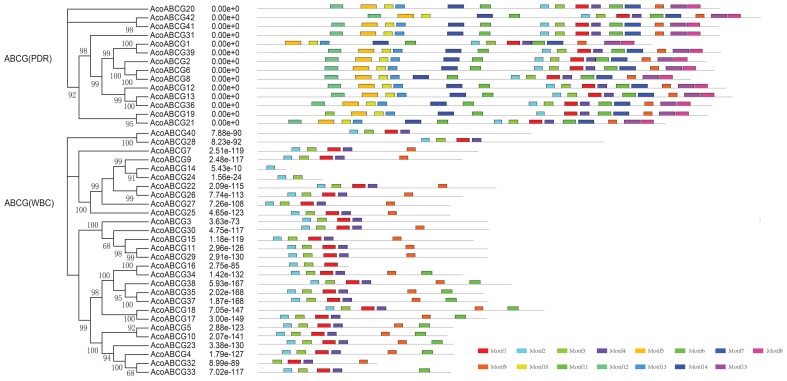
The motif analysis of AcABCG proteins in pineapple. Motifs with specific colors can be find on respective *AcABCG* genes. The combined phylogenetic trees of AcABCG subfamily on the left panel. The motifs of corresponding proteins are shown on the right panel with specific colors on behalf of different motifs using the MEME motif search tool (http://meme-suite.org/tools/meme). The order of the motifs corresponds to their position within individual protein sequences.

To investigate the structure diversity, we analyzed the predicted CDS of individual pineapple *ABC* gene family using Gene Structure Display Server (Guo et al., 2007). The result showed that the exons number of *AcABC* genes varied from 1 to 41. *AcABCB15* had the maximum exons numbers, while seven *AcABC* genes (*AcABCG4*, *AcABCG10*, *AcABCG14*, *AcABCG23*, *AcABCG32*, *AcABCG33* and *AcABCG38*) have only one exon with no UTR (**Figure 2**). To reveal the diversification of *ABCG* subfamily in pineapple, the putative motifs were predicted and identified by MEME with the motif numbers setting from 1 to 15 (Bailey and Elkan, 1994). The *ABCG* subfamily had two types: *PDR* and *WBC*. The numbers of motifs in each *AcPDRs* were approximately twice as many as that of *AcWBCs* (**Figure 3**). *AcABCG14* belonging to *AcWBCs* had only one motif; while most of *AcPDRs* contained 15 motifs except for *AcABCG1*, *AcABCG19*, and *AcABCG42*.

### Expression Profiles of Pineapple *ABC* Genes in Different Tissues

**FIGURE 4:**
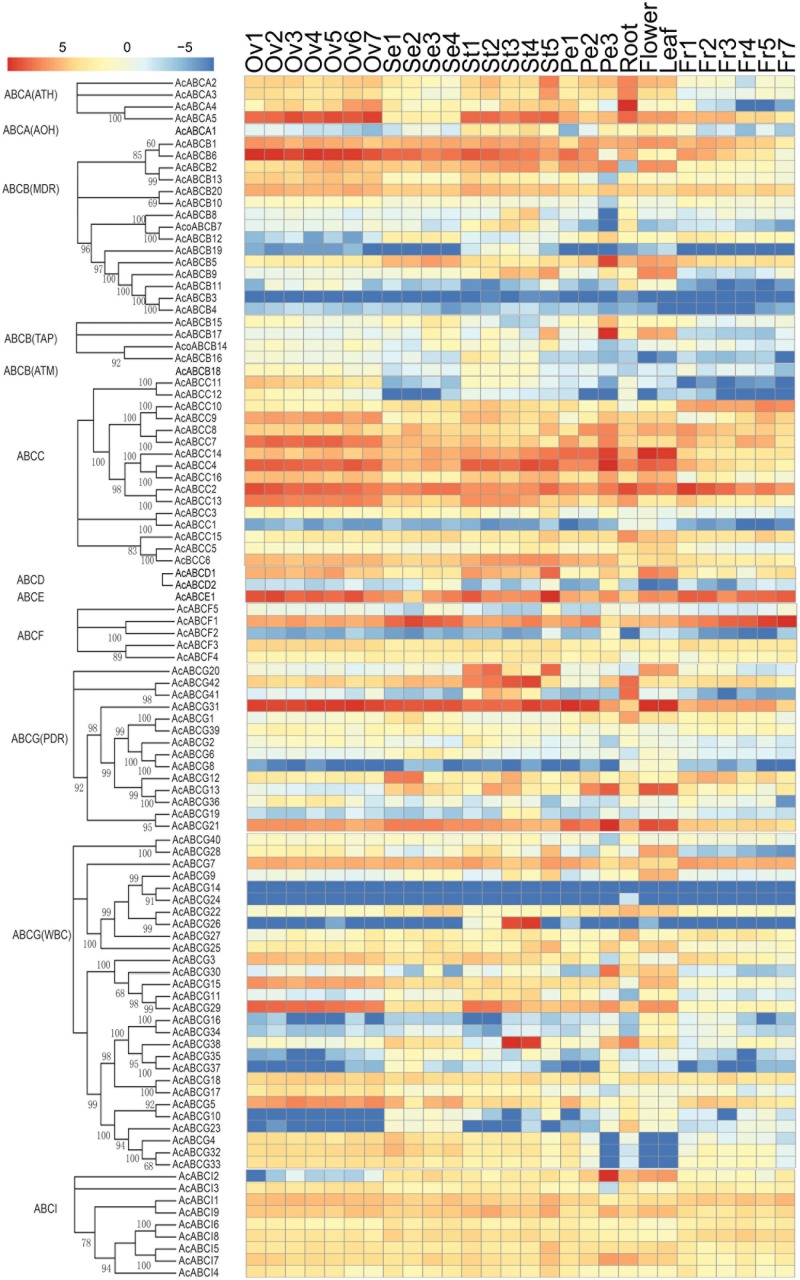
Tissue-specific expression profiles of *AcABC* genes in pineapple. Heat-map of tissue-specific expression profiles of *AcABC* genes in pineapple. RNA-seq expression level can be understood using the given scale and roman numbers on right-side shows hierarchical clusters based on gene expression. The combined phylogenetic trees of pineapple *ABC* family on the left panel. Red color indicates high levels of transcript abundance, and green indicates low transcript abundance. The color scale is shown at the bottom. Samples are mentioned at the top of each lane: ovule S1–S7, sepal S1–S4, stamen S1–S5, petal S1–S3, root, leaf, flower, fruit S1–S7. “S” is abbreviation of word “stage.”

**FIGURE 5:**
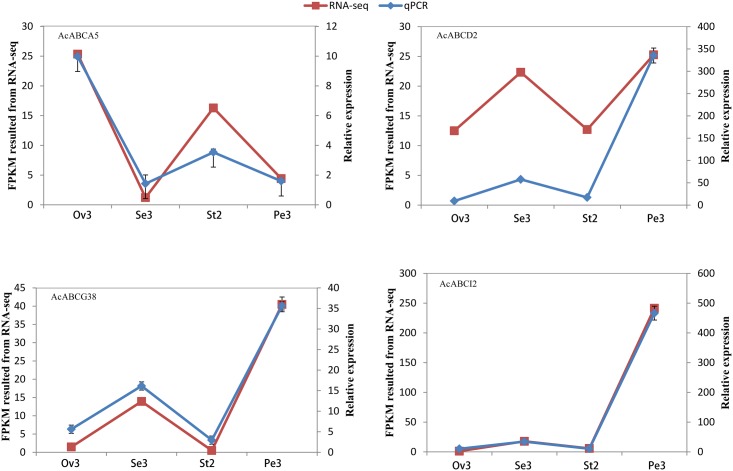
Validation of *AcABC* genes RNA-seq by qRT-PCR analysis of four genes’ expression at five different tissues. Line graphs are constructed from relative gene expression (Left side *Y*-Axis) in different tissues (qRT-PCR) data and FPKM values (RNA-seq) data (Right side *Y*-Axis) for these tissues.

To analyze the expression profiles, the expression level of 100 *AcABC* genes were analyzed based on their FPKM values from RNA-seq data of different tissues. On basis of the average log values of each gene in given tissue, a hierarchical cluster and expression patterns of these genes were generated (**Figure 4**). According to the expression specificity, those *AcABC* genes could be classified into five types. In type I, the genes expressed ubiquitously in most or all tissues such as *AcABCB1*, *AcABCC2*, *AcABCE1*, *AcABCF1* and *AcABCG31*. Conversely, in type II, eleven genes demonstrated comparatively low expression levels in almost all tissues, especially, *AcABCB3*, *AcABCG14* and *AcABCG24* had extremely low expression. Sixty-three genes were moderately and evenly expressed in all organs in type III. The eleven genes in type IV had remarkably high expression levels in particular vegetative and reproductive organs (i.e., *AcABCA5* and *AcABCC4* in ovule and stamen, *AcABCC7*, *AcABCC9*, and *AcABCG29* in ovule). The ten genes were highly and specifically expressed in one or two development stages of organs in type V (*AcABCA4* in root, *AcABCB5*, *AcABCB17* and *AcABCAI2* in petal Pe3, *AcABCC14* and *AcABCG13* in flower and leaf, *AcABCG20* in stamen St1, St2, and St5, *AcABCG42* in stamen St1–St4 as well as *AcABCG26* and *AcABCG38* in stamen St3 and St4) (**Figure 4**). We found some genes, which belong to the same group, had analogical expression profiles. For example, the majority of the *AcABCC* genes were relatively highly expressed in all of tissues, and nearly all of the *AcABCI* members had similar expression levels. To further verify the reliability from RNA-seq data, the expression levels of four genes (*AcABCA5*, *AcABCG38*, *AcABCD2* and *AcABCI2*) in four different tissues were selected for qRT-PCR validation. The results revealed that expression patterns of qRT-PCR were consistent with that from RNA-seq analysis (**Figure 5**).

### *AcABCG38* Might Play Crucial Roles in Pollen and Anther Development

As showed in **Figure 4**, the expression profiling revealed that *AcABC38* expressed highly in stamen St3 and St4, implying that this gene may be involved in stamen development. As previous reports, two *ABC* genes (*AtABCG1* and *AtABCG16*) play significant roles in pollen development (Yadav and Reed, 2014; Yim et al., 2016). Base on phylogenetic analysis, our results showed that *AtABCG1*, *AtABCG16* and *AcABCG38* clustered the same subclade. Those results further indicated *AcABCG38* functions in pollen development. Thereby, we cloned *AcABCG38* gene and transformed into the *Arabidopsis* double mutant *abcg1-2abcg16-2* to check it whether functions or not.

**FIGURE 6:**
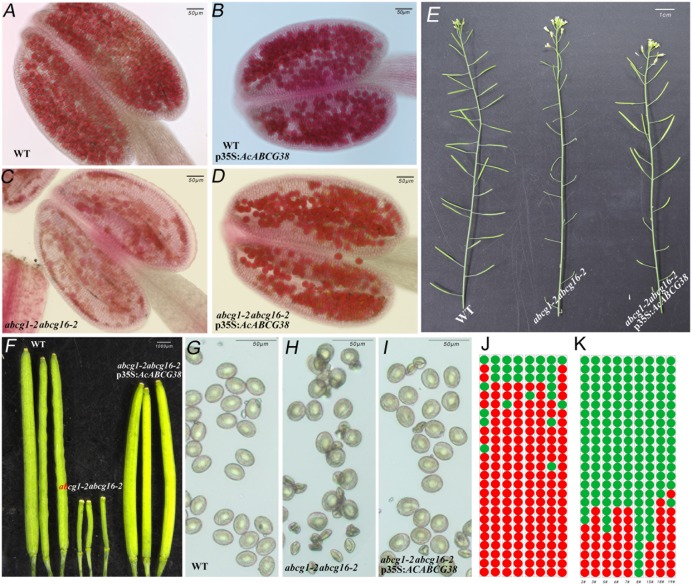
Over-expressing of *AcABCG38* can partly recover the fertility and pollen development defects of double mutant *abcg1-2abcg16-2*. **(A)** Alexander red staining of WT anther. **(B)** Alexander red staining of *p35S:AcABCG38* anther. **(C)** Alexander red staining of double mutant of *abcg1-2abcg16-2* anther. **(D)** Alexander red staining of the *abcg1-2abcg16-2 p35S:AcABCG38* line 8# anther. **(E)** Main branch of the plants with genotype as indicated (left: *col-0*, mid: *abcg1-2abcg16-2*, right: *abcg1-2abcg16-2 p35S:AcABCG3* line 2#). **(F)** Siliques of plants with genotype as indicated (left: *col-0*, mid: *abcg1-2abcg16-2, right:* transgenic plant 8# in *abcg1-2abcg6-2* background). **(G)** Mature pollens of *col-0*. **(H)** Mature pollens of *abcg1-2abcg16-2* plant. **(I)** Mature pollens of the *abcg1-2abcg16-2 p35S:AcABCG38* 8#. **(J)** Seed set phenotype of the 25 siliques from bottom to top in main branch of the *abcg1-2abcg16-2* plants. Red dots represent the short siliques with reduced seed set; green dots represent the normal siliques with full seed set. **(K)** Seed set phenotype of the 25 siliques from bottom to top in main branch of the individual *abcg1-2abcg16-2 p35S:AcABCG38* lines.

As showed in **Figure 6**, the *abcg1-2abcg16-2* double mutant has short siliques, lower seed set (**Figures 6E,F,J**) and collapsed pollens (**Figures 6C,H**). Reciprocal crosses between *abcg1-2abcg16-2* double mutant and wild type showed that the reduced fertility in *abcg1-2abcg16-2* was due to defective male gametophyte function (**Supplementary Table S1**). Moreover, the transmission of *abcg1* allele through pollen but not female reproductive organ was significantly reduced in *abcg16-2/abcg16-2* background (*p* < 0.05) (**Supplementary Table S2**). While the transmission of *abcg16* alleles through both male and female was not affected in *abcg1-2/abcg1-2* background (**Supplementary Table S3**). These results suggested that *AtABCG1* plays dominant role in controlling male gametophytic function.

In the T1 generation of the *p35S:AcABCG38* transgenic plants, we selected nine individual lines that showed elongated siliques (**Figures 6E,F,K**) and less aborted pollens (**Figures 6D,I**) compared with the double mutant *abcg1-2abcg16-2* (**Figures 6C,E,F,H,J** and **Table 2**), however, they had slightly shorter siliques and higher percentage of aborted pollen compared to WT plants (**Figures 6A,E–G** and **Table 2**). These results suggesting that overexpression of *AcABCG38* can partially rescue the reduced fertility and pollen abortion phenotype in *abcg1-2abcg16-2*. The transcript level of *AcABCG38* was also evaluated in individual transgenic lines using qRT-PCR to confirm the complemented phenotype with the increased *AcABCG38* expression. The results showed that complemented line 8# exhibited the highest transcriptional level of *AcABCG38* (**Figure 6K**, **Table 2**, and **Supplementary Figure S2**). Five independent *AcABCG38*-overexpressing transgenic plants in WT background showed comparable vegetative growth to WT plant and full seed set with normal pollens development as indicated by Alexander red staining (**Supplementary Figure S3**). Taken together, these results indicated that *AcABCG38* plays vital roles in pineapple pollen development.

**Table 2:** The *abcg1-2abcg6-2 p35S:AcABCG38* plants have higher percentage of normal pollen than *p35S:AcABCG38* plants.

	WT	*abcg1-2*^-/-^ *abcg16-2*^-/-^	2#	3#	5#	6#	7#	8#	15#	18#	19#
Normal	1331	275	965	656	591	979	478	1044	1418	657	784
Abnormal	3	1015	434	449	476	551	353	275	633	448	393
Total	1334	1290	1399	1105	1066	1530	831	1319	2051	1105	1177
Normal (%)	99.48	21.3	69.0	59.4	55.4	64.0	57.3	79.2	69.1	59.5	66.6


*The nine *abcg1-2abcg6-2 p35S:AcABCG38* transgenic lines (2#, 3#, 5#, 6#, 7#, 8#, 15#, 18#, 19#) in T1 generation were used for pollen phenotype observation. The numbers of pollen grains were counted in culture medium by microscope.*

## Discussion

### Diversity of ABC Transporters in Plants

It is reported that the ABC proteins are relatively conserved across different species but distinct between plants and animals (Xie et al., 2012; Pang et al., 2013). Compared with animal ABC transporters, plant ABC proteins exist numerous and diverse. A number of ABC genes are found in different plant species, e.g., the model plant *Arabidopsis thaliana* contains 129 *ABC* genes, maize 130 ABC transporters and rice 128 transporters (Ponte-Sucre, 2007; Pang et al., 2013). In this study, 100 AcABC proteins have been identified genome-wide. Additionally, those ABC proteins can be divided into as many as eight subfamilies, each subfamily can further be divided into a quantity of subsets (Shao et al., 2013). We also identified 18 *AcABCB* genes, 16 *AcABCC* genes and 42 *AcABCG* genes in pineapple genome, accounting for 76% of all *AcABC* members. Among those subfamilies, *AcABCB* genes includes 13 *AcMDRs*, 4 *AcTAPs* and 1 *AcATMs* in this study. Previous studies reported that *MDRs* are responsible for transporting varieties of substrates, such as lipid proteins, bactericin, peptides, cell surface components, auxin transport and so on (Noh et al., 2001; Sasaki et al., 2002; Moons, 2008). *ATMs* play a vital role in resistance to heavy metals toxicity in plants (Kim et al., 2006). In *Arabidopsis*, two proteins AtMRP4 and AtMRP5 particularly expressed in guard cells and control stomata conductance (Gaedeke et al., 2001; Frelet, 2003). Maize MRP (ZmABCC5), involved in resistance system, also plays role in anthocyanin transport (Goodman et al., 2004). As showed in **Figure 4**, the majority of AcABCC proteins were expressed in all organs in our study, suggesting their potential role in pineapple growth and development. *ABCG* subfamily participates in transporting lipid precursors of cutin and wax (Pighin et al., 2004). *AcABCG* contains 42 members and their expression profiles suggests that either they are highly expressed or specific to tissue. Those data clearly indicate that plants require numerous ABC proteins for growth and development. The reason maybe that plants develop a complicated transporter system to survival in diversity environment through a rapid divergence across a long process of natural evolution and selection.

### *AcABC* Gene Expression Profiles Analysis

Recently, the release of pineapple genome sequence makes it available for researchers to explore pineapple specific agronomic traits (Ming et al., 2015). To explore the function of ABC transporters in pineapple, we downloaded the pineapple different tissues RNA-seq data, and analyzed the expression levels of 100 *AcABC* genes based on their FPKM values. As showed in **Figure 4**, a hierarchical cluster and expression patterns of these genes were generated. Those *AcABC* genes could express specifically. For example, *AcABCB6* expressed in floral organs, *AcABCB5* and *AcABCB17* in Pe3 shows tissue-specific expression. AtABCB1 localized in the plasma membrane and participated in IAA-induced plant development (Sasaki et al., 2002). *AcABCB1*, clustered with *AtABCB1* in same clades by phylogenetic analysis, expressed broadly in all pineapple tissues, suggesting that *AcABCB1* could also be involved in auxin induced pineapple development.

Interesting, some *AcABCs* expressed remarkably in reproductive tissues, e.g., *AcABCA5* and *AcABCC4* in ovule and stamen; *AcABCC7*, *AcABCC9* and *AcABCG29* in ovule; and *AcABCG31* was specific to floral organs; indicating their potential roles in pineapple flower development. *AtABCG1* and *AtABCG16* and redundantly in controlling *Arabidopsis* pollen development (Yadav and Reed, 2014; Yim et al., 2016) and suggests that *AcABCG38* may also be involved in pollen development. The expression pattern of *AcABCs* together with the reported functions of corresponsive *Arabidopsis* orthologs could provide clue to understand the function of *AcABCs* in pineapple.

### *AcABC38* Regulates Pineapple Reproductive Development

Sexual propagation could be an effective and potential way to improve the pineapple agricultural production (Kondrashov, 1993). However, the function of genes in pineapple development are barely reported. Our study aims to identify ABC genes that are crucial for pineapple propagation. For instance, *AcABCG26* and *AcABCG38* were highly expressed in different stages of stamen (**Figure 4**). The expression profiles of these genes in reproductive organs at different developmental stages could determine its functional role. To validate functions of *AcABCs*, *AcABCG38* was selected for functional study as it showed high expression in stamens at St3 and St4. Also *Arabidopsis* homologs of *AcABCG38*, *AtABCG1* and *AtABCG16* are known to work redundantly in pollen development (Yadav and Reed, 2014; Yim et al., 2016).

Over-expression of *AcABCG38* in *Arabidopsis* double mutant *abcg1-2abcg16-2* was able to partly rescue the defective pollen development phenotype, indicating that *AcABCG38* might play important roles for pollen development in pineapple. The mechanism underlying the regulation of *AcABCG38* on pineapple pollen development is still unknown and further studies could shed light on it. Present study of pineapple *AcABC* genes provide useful information on gene function and may act as foundation for future pineapple research.

## Conclusion

The ABC transporter gene families are ubiquitous and important in all kind of life events for all life organism. Whereas the information about pineapple *ABC* gene family is not available. Here we identified 100 *ABC* genes in the pineapple genome and grouped them into eight subfamilies. Gene expression profiling revealed many tissue specific genes particularly in reproductive organs. Furthermore, over-expression of *AcABCG38* in *Arabidopsis* double mutant *abcg1-2abcg16-2* partly rescued the defective pollen development phenotype, indicating that *AcABCG38* might have important function for pollen development in pineapple. Overall, the characterization and expression profile study of pineapple *AcABC* genes provide useful information for gene functional study as well as insights into future pineapple researches for improving pineapple sexual plant reproduction.

## Author Contributions

Created and designed the experiments: PC, YiL, and YQ. Carried out the experiments: PC, LZ, and ZH. The analysis of data: YiL. Contributed reagents/materials/analysis tools: PC, LZ, ZH, MY, BH, YaL, SA, ZZ, ZR, and LL. Wrote the paper: PC and YQ.

## Conflict of Interest Statement

The authors declare that the research was conducted in the absence of any commercial or financial relationships that could be construed as a potential conflict of interest.

## Footnotes

1 https://phytozome.jgi.doe.gov/

2 http://hmmer.org

3 http://www.ncbi.nlm.nih.gov/cdd

4 http://web.expasy.org

5 http://www.ebi.ac.uk/Tools/msa/mafft/

6 http://gsds.cbi.pku.edu.cn

7 http://meme-suite.org/tools/meme

8 http://abrc.osu.edu

9 http://www.qinlab.net

10 https://www.ncbi.nlm.nih.gov/Traces/study/?acc=SRP067011

## Supplementary Material

**FIGURE S1 |** Distribution of *ABC* genes in pineapple genome. *MapChart* was used to located genes on chromosomes. Respective chromosome numbers are indicated at the top of each bar. The bar on the left is respective *ABC* genes. Every bar has different colors, and the length of bar represents the size chromosome in pineapple.

**FIGURE S2 |** Relative expression of *AcABCG38* different transgenic lines in *abcg1-2abcg6-2* background (there are nine transgenic lines for first generation, respectively, lines: 2#, 3#, 5#, 6#, 7#, 8#, 15#, 18#, 19#) and *abcg1-2abcg16-2* (negative control). The result shows that there are consistent with rescued phenotype (i.e., line 8# have detected the highest expression, and it was the most perfectly recovered phenotype line (**Figures 6D,E,K**).

**FIGURE S3 |** Alexander red staining. Five transgenic lines in *col-0* **(A)** and *abcg1-2abcg16-2* background **(B)** respectively.

**TABLE S1 |** Reduced seed-set phenotypes in double mutants *abcg1-2*/*abcg1-2 abcg16-2/abcg16-2* result from defective male reproductive function.

**TABLE S2 |** Transmission of *abcg1* alleles proves through male gametes is significantly deviated from expect in *abcg16-2/abcg16-2* background.

**TABLE S3 |** Transmission of *abcg16* allele proves that *AtABCG16* is not affected in *abcg1-2/abcg1-2* background.

**TABLE S4 |** The primers of vectors construction and qRT-PCR.

**DATASET S1 |** All the AcABC and ATABC protein sequences.

**DATASET S2 |** The used alignments of all the AcABC and ATABC protein sequences.

## References

[B1] Aizen M. A. Harder L. D. (2007). Expanding the limits of the pollen-limitation concept: effects of pollen quantity and quality. *Ecology* 88 271–281. 10.1890/06-1017 17479745

[B2] Arumuganathan K. Earle E. D. (1991). Nuclear DNA content of some important plant species. *Plant Mol. Biol. Rep.* 9 208–218. 10.1007/BF02672069

[B3] Bailey T. L. Elkan C. (1994). Fitting a mixture model by expectation maximization to discover motifs in biopolymers. *Proc. Int. Conf. Intell. Syst. Mol. Biol.* 2 28–36. 7584402

[B4] Cai H. Zhao L. Wang L. Zhang M. Su Z. Cheng Y. (2017). ERECTA signaling controls *Arabidopsis* inflorescence architecture through chromatin-mediated activation of PRE1 expression. *New Phytol.* 214 1579–1596. 10.1111/nph.14521 28295392

[B5] Campa D. Pardini B. Naccarati A. Vodickova L. Novotny J. Forsti A. (2008). A gene-wide investigation on polymorphisms in the ABCG2/BRCP transporter and susceptibility to colorectal cancer. *Mutat. Res.* 645 56–60. 10.1016/j.mrfmmm.2008.08.001 18775442

[B6] Chen F. Zhang X. Liu X. Zhang L. (2017). Evolutionary analysis of MIKC^c^-type MADS-box genes in gymnosperms and angiosperms. *Front. Plant Sci.* 8:895. 10.3389/fpls.2017.00895 28611810 PMC5447709

[B7] Clough S. J. Bent A. F. (1998). Floral dip: a simplified method for *Agrobacterium*-mediated transformation of *Arabidopsis thaliana*. *Plant J.* 16 735–743. 10.1046/j.1365-313x.1998.00343.x 10069079

[B8] Dai X. Bai Y. Zhao L. Dou X. Liu Y. Wang L. (2017). H2A.Z represses gene expression by modulating promoter nucleosome structure and enhancer histone modifications in *Arabidopsis*. *Mol. Plant* 10 1274–1292. 10.1016/j.molp.2017.09.007 28951178

[B9] Davidson A. L. Dassa E. Orelle C. Chen J. (2008). Structure, function, and evolution of bacterial ATP-binding cassette systems. *Microbiol. Mol. Biol. Rev.* 72 317–364. 10.1128/MMBR.00031-07 18535149 PMC2415747

[B10] Eddy S. R. (2011). Accelerated profile HMM searches. *PLOS Comput. Biol.* 7:e1002195. 10.1371/journal.pcbi.1002195 22039361 PMC3197634

[B11] Fassinou Hotegni V. N. Lommen W. J. Agbossou E. K. Struik P. C. (2015). Influence of weight and type of planting material on fruit quality and its heterogeneity in pineapple [*Ananas comosus* (L.) Merrill]. *Front. Plant Sci.* 5:798. 10.3389/fpls.2014.00798 25653659 PMC4300867

[B12] Frelet A. (2003). The plant multidrug resistance ABC transporter AtMRP5 is involved in guard cell hormonal signalling and water use. *Plant J.* 33 119–129. 10.1046/j.1365-313X.2003.016012.x 12943546

[B13] Gaedeke N. Klein M. Kolukisaoglu U. Forestier C. Müller A. Ansorge M. (2001). The *Arabidopsis thaliana* ABC transporter AtMRP5 controls root development and stomata movement. *EMBO J.* 20 1875–1887. 10.1093/emboj/20.8.1875 11296221 PMC125232

[B14] Geisler M. Kolukisaoglu H. U. Bouchard R. Billion K. Berger J. Saal B. (2003). TWISTED DWARF1 a unique plasma membrane-anchored immunophilin-like protein, interacts with *Arabidopsis* multidrug resistance-like transporters AtPGP1 and AtPGP19. *Mol. Biol. Cell* 14 4238–4249. 10.1091/mbc.E02-10-0698 14517332 PMC207015

[B15] Goodman C. D. Casati P. Walbot V. (2004). A multidrug resistance-associated protein involved in anthocyanin transport in *Zea mays*. *Plant Cell* 16 1812–1826. 10.1105/tpc.022574 15208386 PMC514163

[B16] Guo A. Y. Zhu Q. H. Chen X. Luo J. C. (2007). GSDS: a gene structure display server. *Hereditas* 29 1023–1026. 10.1360/yc-007-1023 17681935

[B17] Henderson D. P. Payne S. M. (1994). Vibrio cholerae iron transport systems: roles of heme and siderophore iron transport in virulence and identification of a gene associated with multiple iron transport systems. *Infect. Immun.* 62 5120–5125. 7927795 10.1128/iai.62.11.5120-5125.1994 PMC303233

[B18] Higgins C. F. Linton K. J. (2004). The ATP switch model for ABC transporters. *Nat. Struct. Mol. Biol.* 11 918–926. 10.1038/nsmb836 15452563

[B19] Kim D. Y. Bovet L. Kushnir S. Noh E. W. Martinoia E. Lee Y. (2006). AtATM3 is involved in heavy metal resistance in *Arabidopsis*. *Plant Physiol.* 140 922–932. 10.1104/pp.105.074146 16461380 PMC1400565

[B20] Klein I. Sarkadi B. Váradi A. (1999). An inventory of the human ABC proteins. *Biochim. Biophys. Acta* 1461 237–262. 10.1016/S0005-2736(99)00161-3 10581359

[B21] Kondrashov A. S. (1993). Classification of hypotheses on the advantage of amphimixis. *J. Hered.* 84 372–387. 10.1093/oxfordjournals.jhered.a111358 8409359

[B22] Levitis D. A. Zimmerman K. Pringle A. (2017). Is meiosis a fundamental cause of inviability among sexual and asexual plants and animals? *Proc. Biol. Sci.* 284:20170939. 10.1098/rspb.2017.0939 28768890 PMC5563809

[B23] Ming R. Vanburen R. Wai C. M. Tang H. Schatz M. C. Bowers J. E. (2015). The pineapple genome and the evolution of CAM photosynthesis. *Nat. Genet.* 47 1435–1442. 10.1038/ng.3435 26523774 PMC4867222

[B24] Moons A. (2008). Transcriptional profiling of the PDR gene family in rice roots in response to plant growth regulators, redox perturbations and weak organic acid stresses. *Planta* 229 53–71. 10.1007/s00425-008-0810-5 18830621

[B25] Neiman M. Schwander T. (2011). Using parthenogenetic lineages to identify advantages of sex. *Evol. Biol.* 38 115–123. 10.1007/s11692-011-9113-z

[B26] Noh B. Murphy A. S. Spalding E. P. (2001). Multidrug resistance-like genes of *Arabidopsis* required for auxin transport and auxin-mediated development. *Plant Cell* 13 2441–2451. 10.1105/tpc.13.11.2441 11701880 PMC139463

[B27] Otto S. P. (2009). The evolutionary enigma of sex. *Am. Nat.* 174(Suppl. 1), S1–S14. 10.1086/599084 19441962

[B28] Pang K. Li Y. Liu M. Meng Z. Yu Y. (2013). Inventory and general analysis of the ATP-binding cassette (ABC) gene superfamily in maize (*Zea mays* L.). *Gene* 526 411–428. 10.1016/j.gene.2013.05.051 23747399

[B29] Paponov I. A. Teale W. D. Trebar M. Blilou I. Palme K. (2005). The PIN auxin efflux facilitators: evolutionary and functional perspectives. *Trends Plant Sci.* 10 170–177. 10.1016/j.tplants.2005.02.009 15817418

[B30] Peckham G. D. Kaneshiro W. S. Luu V. Berestecky J. M. Alvarez A. M. (2010). Specificity of monoclonal antibodies to strains of *Dickeya* sp. That cause bacterial heart rot of pineapple. *Hybridoma* 29 383–389. 10.1089/hyb.2010.0034 21050038

[B31] Pighin J. A. Zheng H. Balakshin L. J. Goodman I. P. Western T. L. Jetter R. (2004). Plant cuticular lipid export requires an ABC transporter. *Science* 306 702–704. 10.1126/science.1102331 15499022

[B32] Ponte-Sucre A. (2007). Availability and applications of ATP-binding cassette (ABC) transporter blockers. *Appl. Microbiol. Biotechnol.* 76 279–286. 10.1007/s00253-007-1017-6 17522856

[B33] Price M. N. Dehal P. S. Arkin A. P. (2010). FastTree 2–approximately maximum-likelihood trees for large alignments. *PLOS ONE* 5:e9490. 10.1371/journal.pone.0009490 20224823 PMC2835736

[B34] Pujol C. J. Kado C. I. (2000). Genetic and biochemical characterization of the pathway in *Pantoea citrea* leading to pink disease of pineapple. *J. Bacteriol.* 182 2230–2237. 10.1128/JB.182.8.2230-2237.2000 10735866 PMC111272

[B35] Richard M. Drouin R. Beaulieu A. D. (1998). ABC50 a novel human ATP-binding cassette protein found in tumor necrosis factor-alpha-stimulated synoviocytes. *Genomics* 53 137–145. 10.1006/geno.1998.5480 9790762

[B36] Rodger J. G. Ellis A. G. (2016). Distinct effects of pollinator dependence and self-incompatibility on pollen limitation in South African biodiversity hotspots. *Biol. Lett.* 12:20160253. 10.1098/rsbl.2016.0253 27277954 PMC4938053

[B37] Sánchezfernández R. O. Davies T. G. E. Coleman J. O. D. Rea P. A. (2001). The *Arabidopsis thaliana* ABC protein superfamily, a complete inventory. *J. Biol. Chem.* 276 30231–30244. 10.1074/jbc.M103104200 11346655

[B38] Santos C. Ventura J. A. Lima N. (2016). New insights for diagnosis of pineapple fusariosis by MALDI-TOF MS technique. *Curr. Microbiol.* 73 206–213. 10.1007/s00284-016-1041-9 27117163

[B39] Sasaki T. Ezaki B. Matsumoto H. (2002). A gene encoding multidrug resistance (MDR)-like protein is induced by aluminum and inhibitors of calcium flux in wheat. *Plant Cell Physiol.* 43 177–185. 10.1093/pcp/pcf025 11867697

[B40] Schneider E. Hunke S. (1998). ATP-binding-cassette (ABC) transport systems: functional and structural aspects of the ATP-hydrolyzing subunits/domains. *FEMS Microbiol. Rev.* 22 1–20. 10.1111/j.1574-6976.1998.tb00358.x 9640644

[B41] Shao R. Shen Y. Zhou W. Fang J. Zheng B. (2013). Recent advances for plant ATP-binding cassette transporters. *J. Zhejiang A F Univ.* 30 761–768.

[B42] Su Z. Wang L. Li W. Zhao L. Huang X. Azam S. M. (2017). Genome-wide identification of auxin response factor (ARF) genes family and its tissue-specific prominent expression in pineapple (*Ananas comosus*). *Trop. Plant Biol.* 10 86–96. 10.1007/s12042-017-9187-6

[B43] Trapnell C. Roberts A. Goff L. Pertea G. Kim D. Kelley D. R. (2012). Differential gene and transcript expression analysis of RNA-seq experiments with TopHat and Cufflinks. *Nat. Protoc.* 7 562–578. 10.1038/nprot.2012.016 22383036 PMC3334321

[B44] Tyzack J. K. Wang X. Belsham G. J. Proud C. G. (2000). ABC50 interacts with eukaryotic initiation factor 2 and associates with the ribosome in an ATP-dependent manner. *J. Biol. Chem.* 275 34131–34139. 10.1074/jbc.M002868200 10931828

[B45] Verrier P. J. Bird D. Burla B. Dassa E. Forestier C. Geisler M. (2008). Plant ABC proteins–a unified nomenclature and updated inventory. *Trends Plant Sci.* 13 151–159. 10.1016/j.tplants.2008.02.001 18299247

[B46] Wang W. Zhang L. Guo N. Zhang X. Zhang C. Sun G. (2014). Functional properties of a cysteine proteinase from pineapple fruit with improved resistance to fungal pathogens in *Arabidopsis thaliana*. *Molecules* 19 2374–2389. 10.3390/molecules19022374 24566309 PMC6271751

[B47] Xie X. Cheng T. Wang G. Duan J. Niu W. Xia Q. (2012). Genome-wide analysis of the ATP-binding cassette (ABC) transporter gene family in the silkworm, *Bombyx mori*. *Mol. Biol. Rep.* 39 7281–7291. 10.1007/s11033-012-1558-3 22311044

[B48] Yadav V. Reed J. W. (2014). ABCG transporters are required for suberin and pollen wall extracellular barriers in *Arabidopsis*. *Plant Cell* 26 3569–3588. 10.1105/tpc.114.129049 25217507 PMC4213157

[B49] Yim S. Khare D. Kang J. Hwang J. U. Liang W. Martinoia E. (2016). Postmeiotic development of pollen surface layers requires two *Arabidopsis* ABCG-type transporters. *Plant Cell Rep.* 35 1863–1873. 10.1007/s00299-016-2001-3 27271688

[B50] Zhang C. Wang D. Yang C. Kong N. Shi Z. Zhao P. (2017). Genome-wide identification of the potato WRKY transcription factor family. *PLOS ONE* 12:e0181573. 10.1371/journal.pone.0181573 28727761 PMC5519183

[B51] Zhao Z. Fang L. L. Johnsen R. Baillie D. L. (2004). ATP-binding cassette protein E is involved in gene transcription and translation in *Caenorhabditis elegans*. *Biochem. Biophys. Res. Commun.* 323 104–111. 10.1016/j.bbrc.2004.08.068 15351708

