# Supplementary figures and images for: Genome-Wide Identification and Expression Profiling of ATP-Binding Cassette (ABC) Transporter Gene Family in Pineapple (Ananas comosus (L.) Merr.) Reveal the Role of AcABCG38 in Pollen Development
Source: Front Plant Sci. 2017 Dec 19;8:2150. doi: 10.3389/fpls.2017.02150 (PMC5742209; doi:10.3389/fpls.2017.02150)

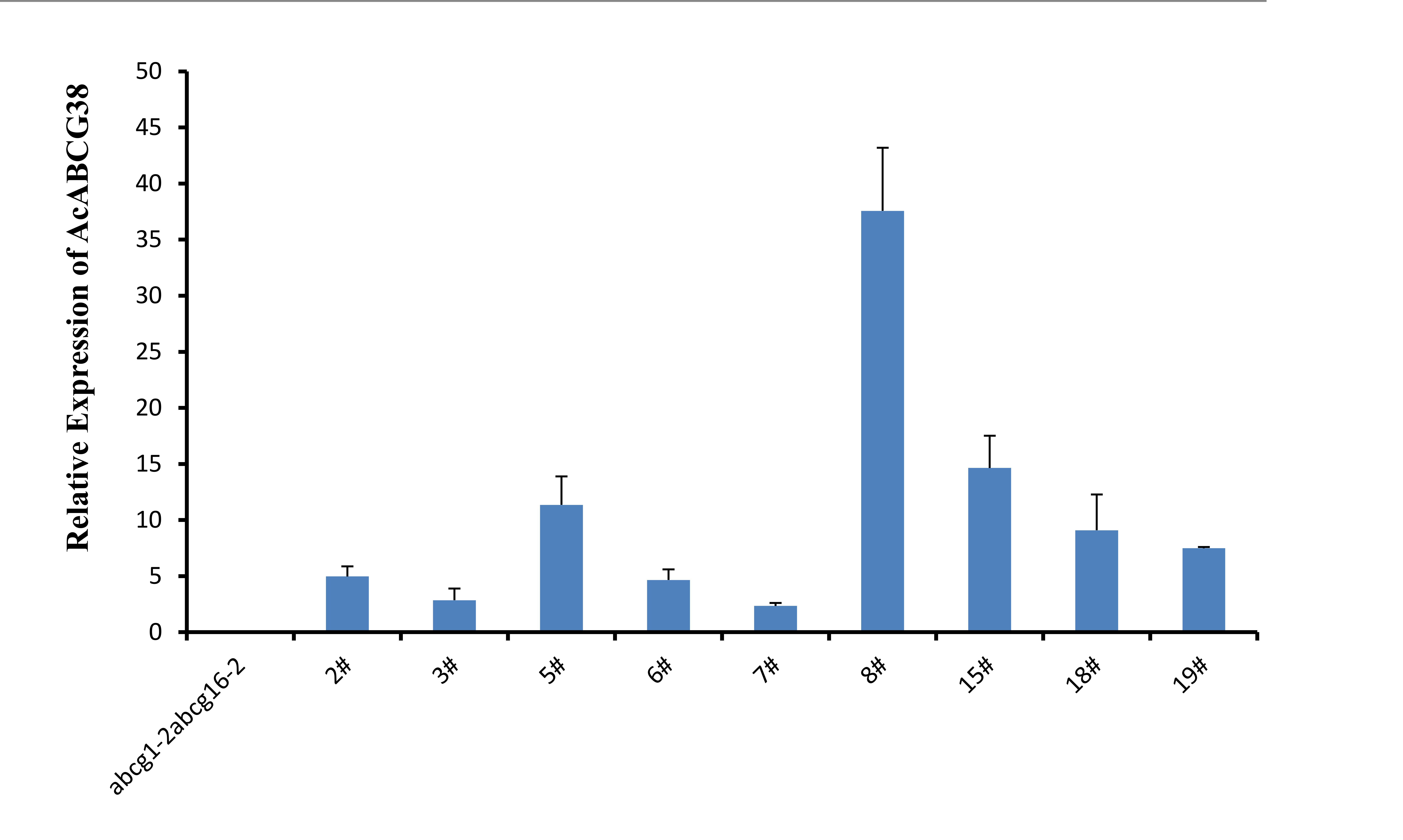

Supplement: Supplementary file 2 [file Image_2.TIF]

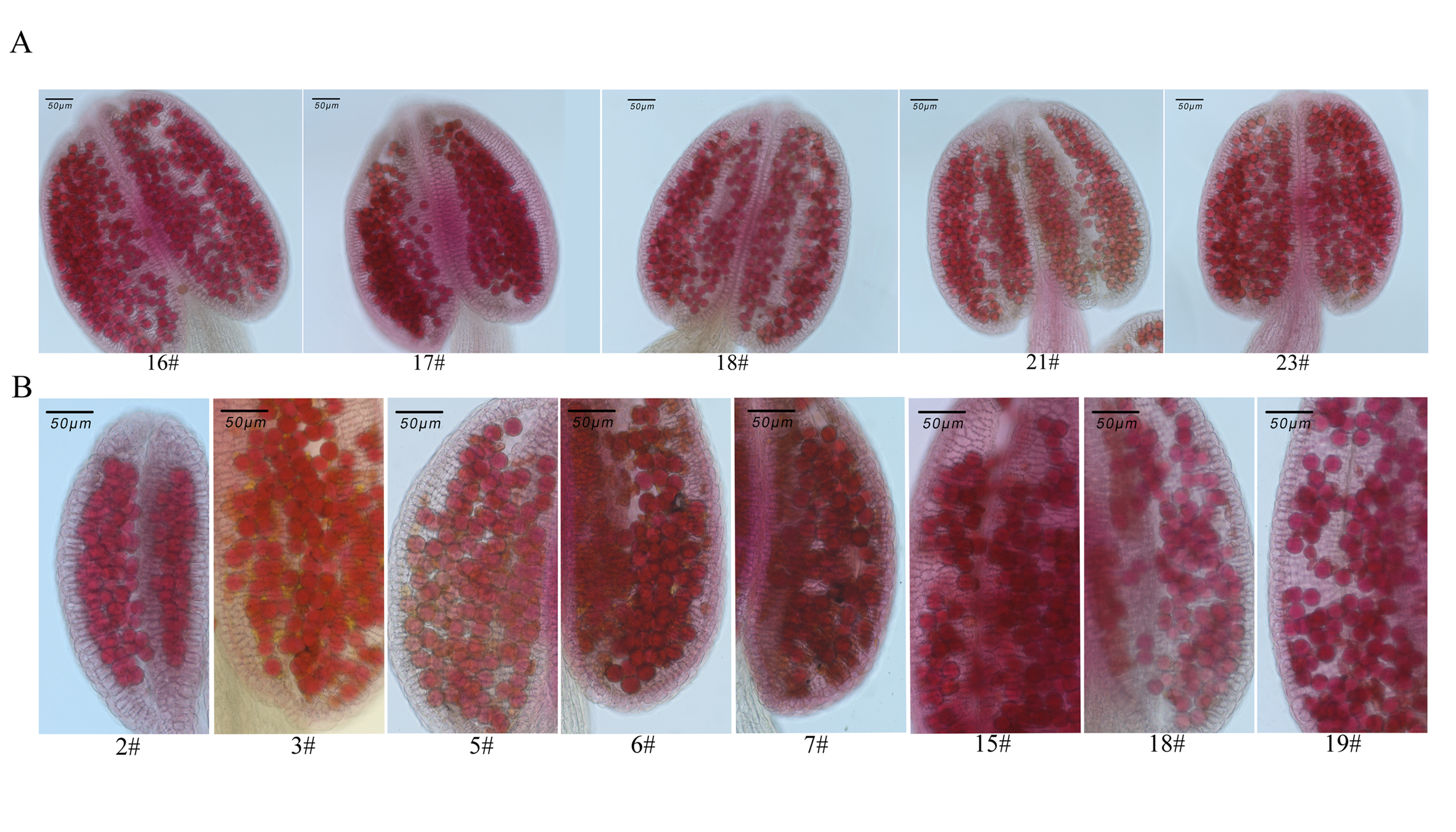

Supplement: Supplementary file 3 [file Image_3.TIF]
