# Supplementary material for: Genome-Wide Identification and Expression Profiling of ATP-Binding Cassette (ABC) Transporter Gene Family in Pineapple (Ananas comosus (L.) Merr.) Reveal the Role of AcABCG38 in Pollen Development
Source: Front Plant Sci. 2017 Dec 19;8:2150. doi: 10.3389/fpls.2017.02150 (PMC5742209; doi:10.3389/fpls.2017.02150)
Supplement: Supplementary file 4 [file Table_1.DOC]

**Table S1.** Reduced seed-set phenotypes in double mutants *abcg1-2/ abcg1-2 abcg16-2/ abcg16-2* result from defective male reproductive function.

| Female parent | Male parent | Pollination | Developed seeds (%) | Siliques counted (n) |
| --- | --- | --- | --- | --- |
| *abcg1-2/ abcg1-2 abcg16-2/ abcg16-2* | *abcg1-2/ abcg1-2 abcg16-2/ abcg16-2* | Auto self | 59.4 | 5 |
| *abcg1-2/ abcg1-2 abcg16-2/ abcg16-2* | *ABCG1/ ABCG 1 ABCG16/ABCG16* | Manual self | 97.7 | 18 |
| *ABCG1/ ABCG 1 ABCG16/ABCG16* | *ABCG1/ ABCG 1 ABCG16/ABCG16* | Manual self | 96 | 5 |
| *ABCG1/ ABCG 1 ABCG16/ABCG16* | *abcg1-2/ abcg1-2 abcg16-2/ abcg16-2* | Manual self | 61.7 | 7 |

|  |
| --- |
|  |

|  |  |  |
| --- | --- | --- |
|  |  |

|  |  |  |
| --- | --- | --- |
|  |  |

|  |  |  |
| --- | --- | --- |
|  |  |
