# Supplementary material for: Genome-Wide Identification and Expression Profiling of ATP-Binding Cassette (ABC) Transporter Gene Family in Pineapple (Ananas comosus (L.) Merr.) Reveal the Role of AcABCG38 in Pollen Development
Source: Front Plant Sci. 2017 Dec 19;8:2150. doi: 10.3389/fpls.2017.02150 (PMC5742209; doi:10.3389/fpls.2017.02150)
Supplement: Supplementary file 5 [file Table_2.DOC]

**Table S2.** Transmission of abcg1 alleles proves through male gametes is significantly deviated from expect in *abcg16-2 /abcg16-2* background.

| **Parental Genotype** | | **Segregation of *abcg1* Allele in Progeny** | | |
| --- | --- | --- | --- | --- |
| **Female** | **Male** | **+/+** | **+/-** | **p-val** |
| *abcg1-2/ABCG1-2 abcg16-2/abcg16-2* | *ABCG1/ ABCG 1 ABCG16/ABCG16* | 124 | 121 | 0.848 |
| *ABCG1/ ABCG 1 ABCG16/ABCG16* | *abcg1-2/ABCG1-2 abcg16-2/abcg16-2* | 176 | 112 | 1.62E-4 |

X^2^ test for an expected segregation ratio of 1:1.

+, wild-type allele; -, mutant allele;

|  |  |  |
| --- | --- | --- |
|  |  |

|  |  |  |
| --- | --- | --- |
|  |  |

|  |  |  |
| --- | --- | --- |
|  |  |
