# Supplementary material for: Genome-Wide Identification and Expression Profiling of ATP-Binding Cassette (ABC) Transporter Gene Family in Pineapple (Ananas comosus (L.) Merr.) Reveal the Role of AcABCG38 in Pollen Development
Source: Front Plant Sci. 2017 Dec 19;8:2150. doi: 10.3389/fpls.2017.02150 (PMC5742209; doi:10.3389/fpls.2017.02150)
Supplement: Supplementary file 7 [file Table_4.doc]

**Table S4.** The primers of vectors construction and qRT-PCR

| **Primer Names** | **Primer Sequences** | Purpose |
| --- | --- | --- |
| |  | | --- |   SALK_087501 -1 | GTCAATAAAACCCATTTCGCC | mutant genotyping |
| SALK_087501 -2 | ACTTCTCGGGAGACGAAACTC | mutant genotyping |
| SALK_055389-1 | GTTTGAGCTATCTCCGGATCC | mutant genotyping |
| SALK_055389-2 | CTCTTCTTTAAAAGCGACGGG | mutant genotyping |
| SALK_087501-1 | ATCCCATACGTCAATTCTCCC | mutant genotyping |
| SALK_087501-2 | ACGTGGACACACCGTCTTTAG | mutant genotyping |
| SALK_119868-1 | TTCTTCCACAATCTCAATGCC | mutant genotyping |
| SALK_119868-2 | CAGACGCTCAATCCTCAACTC | mutant genotyping |
| LBb1.3 | ATTTTGCCGATTTCGGAAC | mutant genotyping |
| AcABCG38-1 | CACCATGTCGCGGTTCGTGGACAAGCTCT | vector construction |
| AcABCG38 -2 | CCTCCTCTTGTTCTTGCTCCCAAGCAGC | vector construction |
| AtABCG1-1 | AGAACAGAGGATCCGGAGATAG | qRT-PCR |
| AtABCG 1-2 | CGCCTAGAACAGCCATGATT | qRT-PCR |
| AtABCG 16-1 | GCTGGAGGAACAAGAGGATTAG | qRT-PCR |
| AtABCG 16-2 | CGGAGTTAGGGTTTGAGGATTG | qRT-PCR |
| AcAcABCA5-1 | GGAGCGAGAGGTAGAGAATTTAG | qRT-PCR |
| AcAcABCA5-2 | GGCGATTCTGACTTCTTGTTTAG | qRT-PCR |
| AcAcABCD2-1 | GGTCACCGAACGACACTTTA | qRT-PCR |
| AcAcABCD2-2 | CCAGTTACGACCACCACTATC | qRT-PCR |
| AcAcABCG38-1 | CAGGCCATTACCGATCTCAATA | qRT-PCR |
| AcAcABCG38-2 | GCAGCAATGCAAAGTAGAAGAG | qRT-PCR |
| AcAcABCI2-1 | CTCACAGCGTGAAGCAGAT | qRT-PCR |
| AcAcABCI2-2 | GTTGTGCGTGGGACAAATTC | qRT-PCR |

|  |
| --- |
|  |

|  |  |  |
| --- | --- | --- |
|  |  |

|  |  |  |
| --- | --- | --- |
|  |  |

|  |  |  |
| --- | --- | --- |
|  |  |
